# Protective Immunity Induced by DNA Vaccines Encoding TgGRA47 and TgGRA72 Against *Toxoplasma gondii* Infection in BALB/c Mice

**DOI:** 10.1155/tbed/9513737

**Published:** 2026-06-05

**Authors:** Zihan Zhou, Jingqi Mu, Jie Sun, Jia Chen, Chenhui Zhou

**Affiliations:** ^1^ Department of Neurosurgery, Ningbo Key Laboratory of Nervous System and Brain Function, The First Affiliated Hospital of Ningbo University, Ningbo, 315010, Zhejiang, China, nbu.edu.cn; ^2^ Health Science Center, Ningbo University, Ningbo, Zhejiang, China, nbu.edu.cn

**Keywords:** DNA vaccine, protective immunity, TgGRA47, TgGRA72, *Toxoplasma gondii*

## Abstract

*Toxoplasma gondii* (*T. gondii*) is a protozoan parasite that lives inside cells and causes zoonotic diseases in the vast majority of homeothermic vertebrates, such as humans. Despite the critical biological roles of TgGRA47 and TgGRA72 in the determination of *T. gondii* growth and virulence, no study has been focused on the possibility of whether these two dense granule proteins (GRAs) could be used as DNA vaccine candidate against *T. gondii* infection. To tackle this issue, we assessed the immunity protection provided by DNA vaccines pVAX‐GRA47 and pVAX‐GRA72 in BALB/c mice infected with the *T. gondii* PRU strain. Our findings demonstrate that TgGRA47 and TgGRA72 are potential DNA vaccine candidates, with the ability to generate strong humoral as well as Th1‐ and Th17‐polarized cellular protective immunity against toxoplasmosis in mouse models.

## 1. Introduction

*Toxoplasma gondii* (*T. gondii*) is a protozoan parasite that lives inside cells and causes zoonotic diseases in the vast majority of homeothermic vertebrates, such as humans [1, 2]. Commonly, *T. gondii* infections are without clinical signs in immunologically healthy hosts, but it could lead to severe toxoplasmosis in immunocompromised hosts, including those with organ transplants, cancer, or AIDS [3, 4]. Also, infection potential occurs in pregnant women, leading to congenital infection in their unborn fetuses [5]. In animals, *T. gondii* can still infect a broad range of hosts, including domestic animals; in particular, it could lead to neonatal loss, stillbirth, and abortion in pigs and sheep, posing with serious economic losses and major public health challenges [6, 7].

Given the severe consequences induced by toxoplasmosis in the hosts, effective treatment or prevention alternatives are essential. Current chemical drugs are mainly involved in pyrimethamine and sulfadiazine, which are used to combat acute *T. gondii* infections, but they could not eliminate tissue cysts [8, 9]. Also, these drug treatments may cause some concerns including drug residues and the development of drug‐resistant parasites [10]. Therefore, immunoprophylaxis is considered a viable approach for preventing *T. gondii* infection. However, the only commercial vaccine, a live‐attenuated vaccine based on tachyzoite S48 strain (Toxovax), was used for protecting against abortion in sheep, but it could not be further used in humans clinically due to the limitations of reversion to the pathogenic phenotype [11]. Hence, it is essential to create an effective and safe vaccine for protection against toxoplasmosis. Significant advances have been achieved in the development of DNA vaccines due to their advantages of safety, convenience, affordability, and their capacity to provoke robust antibody‑mediated and cell‑mediated immune responses against intracellular pathogens [12, 13]. While many *T. gondii* antigens, such as dense granule proteins (GRAs), microneme proteins (MICs), rhoptry proteins (ROPs), and surface antigens (SAGs), have been investigated as DNA vaccine candidates, identifying antigens that effectively stimulate protective cellular immunity remains an ongoing challenge [14, 15]. Generally, GRAs play a crucial role in invading, forming parasite vacuoles, manipulating host cells, and evading host immune responses [16], but also these GRAs are usually explored to be used as potential vaccine candidates in animal models [15]. Among the GRAs, *Toxoplasma* dense GRAs GRA47 (TgGRA47) and GRA72 (TgGRA72) have recently been identified to have a major impact in modulating small‐molecule permeability of the parasitophorous vacuole membrane [17]. Furthermore, GRA47 is also characterized to be required for the proliferation of *Toxoplasma* and also to be important for acute and chronic virulence in mouse models [18]. These critical biological roles of GRA47 and GRA72 in the determination of *T. gondii* growth and virulence have proposed a potentiality that these two dense GRAs could be used as DNA vaccine candidates to combat *T. gondii* infection. However, no investigations have been devoted to study the potentiality of the DNA vaccine encoding TgGRA47 and TgGRA72.

Hence, this study sought to build the eukaryotic plasmids, pVAX‐GRA47 and pVAX‐GRA72, and then to determine the immune responsive efficacies induced by DNA immunization with pVAX‐GRA47 and/or pVAX‐GRA72 and to measure the protective potential of these two DNA vaccine candidates targeting both active and latent *T. gondii* infection in murine models.

## 2. Methods

### 2.1. Parasites and Mice

Zhejiang’s Experimental Animal Facility in Hangzhou, China, provided 6‐ to 8‐week‐old female BALB/c and Kunming mice, which were maintained under specific pathogen‐free (SPF) conditions in line with the Animal Ethics Regulations. Ethical approval for this study was granted by the Ethics Committee of Ningbo University.

The ME49 strain tachyzoites (Type II) were kept in our laboratory and cultured in confluent monolayers of human foreskin fibroblasts (HFFs) (HS27; ATCC: CRL‐1634) using DMEM (Invitrogen, CA, USA) at 37°C and 5% CO_2_ supplemented with 10% fetal bovine serum (FBS, Gibco, MA, USA), and includes 2 mM glutamine, 100 U/mL penicillin, and 10 μg/mL streptomycin as per previously described methods [19]. According to previously outlined methods, the tachyzoites obtained served to generate *T. gondii* lysate antigen (TLA) [20]. Cysts from the PRU strain were preserved and collected from the brains of Kunming mice a month after they were given 20 cysts orally, as described earlier [21]. In brief, tissue cysts were obtained from the brains of chronically infected mice and used for subsequent infections. Brain homogenates were prepared, and cysts were counted under a light microscope to determine the required number for infection. To preserve the biological characteristics of the parasite, including cyst‐forming ability and virulence stability, the number of passages was kept to a minimum.

HEK 293‐T cells (derived from human embryonic kidney) were maintained under standard culture conditions similar to those used for HFF. They were cultivated in DMEM supplemented with 10% FCS (containing 100 IU/mL streptomycin and 100 IU/mL penicillin) at 37°C in a 5% CO2. These 293‐T cells were further used for transfection.

### 2.2. Construction of Plasmids pVAX‐GRA47 and pVAX‐GRA72

RNA was extracted from the *T. gondii* ME49 strain using the TRI reagent (Sigma, MO, USA), and cDNA was synthesized through reverse transcription with the SuperScript First‐Strand Synthesis Kit (Invitrogen, CA, USA). To construct pVAX I ‐HA ‐expressing plasmids for GRA47 and GRA72, the coding sequences of TgGRA47 (Gene ID: 29769301) and TgGRA72 (Gene ID: 7897372) were obtained via PCR‐based amplification from ME49 tachyzoite cDNA with gene‑specific primers (GRA47, forward primer: 5’‐ CCGGAATTCATGCTCCAGATGGCACGATATA‐3’; GRA47, reverse primer: 5’‐GCTCTAGATTAATTACCCTTAGTGGGTGGTTT‐3’; GRA72, forward primer: 5–CCGGAATTCATGGCCATGCGCTCCCGACT–3; and GRA72, reverse primer: 5–GCTCTAGA CACCGAATCAATCGACGCCG–3), where *Kpn*I and *Xba*I restriction sites were incorporated. Followed by ligation, the PCR product with the pMD‐18T Vector (TaKaRa, China; Cat. Number 6011), pMD‐GRA47, and pMD‐GRA72 was generated. After being released from pMD‑GRA47 or pMD‑GRA72 via *Kpn*I/*Xba*I digestion, the GRA47 and GRA72 fragments were individually inserted into pVAX I‑HA (Invitrogen, CA, USA) that had previously been cleaved with *Kpn*I and *Xba*I. PCR and double restriction enzyme cleavage were performed to verify the recombinant DNA plasmids pVAX‐GRA47 and pVAX‐72. Positive clones were then sent for DNA sequencing. Using anion‐exchange chromatography, plasmids that tested positive were harvested from transformed DH5α *E. coli* bacteria, as per the protocol provided by the commercial endotoxin‑free plasmid Giga preparation kit (Qiagen, MD, USA). Plasmid purity and concentration were determined by measuring the absorbance at wavelengths of 260 and 280 nm. Plasmid samples were maintained at –20°C pending use.

### 2.3. Bioinformatic Prediction and *In Vitro* Expression of GRA47 and GRA72

To evaluate the immunogenic potential of TgGRA47 and TgGRA72, bioinformatic analyses were performed using the PROTEAN module of DNASTAR software. To forecast potential B‐cell epitopes and antigenic regions within protein sequences, the hydrophilicity score, antigenicity index, regions of flexibility, and probability of surface localization were examined. For the purpose of evaluating the expression of recombinant plasmids in vitro, pVAX‐GRA47 and pVAX‐GRA72 were introduced into 293‐T cells with Lipofectamine 2000 (Invitrogen, CA, USA) based on the manufacturer’s protocol. We followed the methods of our previous work [22]. In brief, 48 h after transfection with plasmids, the cells were fixed with cool acetone for 30 min, and then they were washed by PBS‐0.1% Triton‐X‐100 (PBST) three times. Subsequently, the cells were treated with polyclonal antibody directed against the HA tag (Proteintech, IL, USA) at 37°C for 1 h and washed with PBST, followed by FITC‐labeled goat‐antirabbit IgG (Proteintech, IL, USA) antibody diluted 1:100 in PBST at room temperature for 45 min in the dark. The nuclei were counterstained with DAPI for 5 min. Finally, the coverslips were mounted using antifade mounting medium, and the specific fluorescence was examined using a Zeiss Axio‐plan fluorescence microscope (Carl Zeiss, Jena, Germany). 293‐T cells transfected with empty pVAX I served as the negative control. Representative images were acquired with appropriate filter sets, and merged images and brightfield references were included to visualize cellular morphology. Scale bars were added to all images.

To confirm recombinant protein expression, 293‐T cells were introduced with pVAX‐GRA47, pVAX‐GRA72, or the empty pVAX I vector via Lipofectamine 2000 (Invitrogen, CA, USA) following the product guidelines. Forty‐eight hours after transfection, the cells were pelleted and then resuspended in RIPA buffer for cell lysis (Beyotime, Shanghai, China). Lysis proceeded on ice for 30 min. Upon centrifugation, the liquid phase was collected from the lysates and used for protein analysis, with protein concentrations determined using a BCA Protein Assay Kit (Thermo Fisher Scientific, MA, USA). Equal amounts of protein samples were separated by 10% SDS‐polyacrylamide gel electrophoresis (SDS‐PAGE) and subsequently transferred onto polyvinylidene fluoride (PVDF) membranes (Millipore, MA, USA). The membranes were blocked with 5% nonfat milk in TBST for 1 h at room temperature and then incubated overnight at 4°C with HA tag polyclonal antibody (dilution 1:1000; Proteintech, IL, USA). Following three washes with TBST, membranes were incubated with horseradish peroxidase (HRP)–conjugated goat antirabbit IgG secondary antibody (Proteintech, IL, USA) for 1 h at room temperature. Protein bands were then detected using an enhanced chemiluminescence (ECL) system (Thermo Fisher Scientific, MA, USA) according to the manufacturer’s protocol.

### 2.4. DNA Vaccination and *T. gondii* Challenge

We followed the methods of our previous work [22]. A total of six groups (a group of 20 mice) were utilized in this investigation. Experimental groups were intramuscularly injected (three times at 2‐week intervals) with pVAX‐GRA47 (100 μL; 1 mg/mL), pVAX‐GRA72 (100 μL; 1 mg/mL), or pVAX‐GRA47 + pVAX‐GRA72 (100 μL; 1 mg/mL), respectively. Control groups received pVAX I (100 μL; 1 mg/mL), PBS (100 μL), or blank control (no treatment) by intramuscular injection using the same schedule. Blood was collected from the tail vein of mice in each group prior to immunization at 0, 2, 4, and 6 weeks. The serum was obtained by centrifugation at 4000 × g for 5 min and subsequently kept at –20°C until later analysis. A total of 8 mice in each group were given oral infected with 100 cysts of the PRU line of *T. gondii* strain 2 weeks following the booster vaccination, and their survival duration was documented daily until death occurred. A further six mice per group were exposed to a nonlethal inoculum of 20 *T. gondii* PRU cysts, and the brain cyst count was evaluated 30 days later according to an established method [16]. Briefly, a glass tissue grinder was employed to homogenize the brains in 1 mL of PBS. A 10 μL aliquot of the homogenate was placed on a microscope slide and examined with a standard light microscope. The number of cysts was counted in three independent aliquots, and the average value was multiplied by the dilution factor to calculate the total cyst number per brain. In the meanwhile, six mice from each group were euthanized, and their splenocytes were collected for lymphocyte proliferation assay, flow cytometric analysis, and determination of cytokines and cytotoxic T lymphocyte (CTL) responses 14 days after the last vaccination.

### 2.5. Antibody Analysis

Serum IgG antibodies and IgG isotypes were detected via an ELISA method as previously described [23]. Serum samples were collected from the tail vein of mice in all groups at 0, 2, 4, and 6 weeks after immunization. Briefly, 100 μL of TLA (10 μg/mL) was transferred to each well of a 96‑well plate and kept at 4°C overnight for incubation. Serum samples were diluted in PBS at a fixed dilution of 1:100 and added to the plates, followed by incubation for 1 h at room temperature. After washing with PBST, HRP‐conjugated goat antimouse IgG, IgG1, or IgG2a antibodies (Sigma–Aldrich, MO, USA) were added and incubated for 60 min at 37°C. The plates were washed with PBST, followed by adding 100 μL of substrate solution and kept for 30 min to detect immune complexes. Absorbance values were read at 405 nm with an ELISA microplate reader (Bio‐Tek EL × 800, VT, USA). Antibody levels were expressed as OD405 values obtained at the fixed serum dilution (1:100). Triplicate measurements were performed for every sample.

### 2.6. MTT‑Based Analysis of Lymphocyte Proliferation

As reported earlier, spleen cells were prepared from three mice in every group at 2 weeks following the last vaccination [21]. Lysis buffer was applied to eliminate erythrocytes. Subsequently, the spleen cells were resuspended in DMEM plus 10% FCS. In brief, 2 × 10^6^ spleen cells in every well were placed in 96‐well plates and cultured with concanavalin A (ConA) (5 μg/mL; Sigma, MO, USA), culture medium alone, or TLA (10 μg/mL) at 37°C under 5% CO_2_ for 72 h. Subsequently, each well received 10 µL of 3‐(4,5‐dimethylthiazol‐2‐yl)‐2,5‐diphenyltetrazolium bromide (MTT, 5 mg/mL, Sigma, St. Louis, MO, USA) was added to each well and incubated for 4 h. The stimulation index (SI) was calculated by using the formula: SI = (OD570TLA/OD570Control):(OD570ConA/OD570Control). Triplicate measurements were performed for every sample.

### 2.7. Flow Cytometric Detection

We followed the methods of our previous work [22]. Initial gating of lymphocytes was performed according to forward and side scatter characteristics. CD3^+^ T cells were identified and further gated into CD4^+^ and CD8^+^ T‐cell subsets. Dendritic cells (DCs) were defined as CD11c^+^ cells, followed by analysis of CD83, CD86, MHC‐I, and MHC‐II expression. Isotype control antibodies were used to exclude nonspecific binding. To analyze the proportions of CD4^+^ and CD8^+^ T cells, the splenocyte suspensions (a total of 1 × 10^6^ purified splenic lymphocytes in the mice in each group) were incubated with PE‐labeled anti‐CD3 (BioLegend, CA, USA, U), APC‐labeled anti‐CD4 (BioLegend, CA, USA), and FITC‐labeled anti‐CD8a (eBioscience, CA, USA) for 30 min at 4°C while protected from light and then fixed with FACScan buffer and 2% paraformaldehyde.

To evaluate CD83, CD86, and MHC molecule expression on splenic DCs, the obtained cells were stained with CD11c‐FITC (BioLegend, California, United States; Cat. Number 117305), CD83‐PE (BioLegend, CA, USA), CD11c‐FITC CD86‐PE (eBioscience, CA, USA), CD11c‐FITC MHC‐I‐PE (eBioscience, CA, USA), and CD11c‐FITC MHC‐II‐PE (eBioscience, CA, USA) in darkness at 4°C for half an hour. Samples were analyzed for fluorescence using a FACScan flow cytometer (BD Biosciences, CA, USA) and processed with SYSTEM II software (Coulter). All measurements were performed in triplicate for samples obtained from six individual mice.

### 2.8. Determination of Cytokine In Vitro

So as to detect specific immune responses against *T. gondii*, the cytokines from splenocytes stimulated with TLA were used for detection. Also, to further delineate DNA immunization‐induced immune memory, splenic lymphocytes were collected 4 weeks after the third immunization as described for the lymphocyte proliferation assay and then cultured with medium alone (negative control) or TLA (10 mg/mL) in corresponding wells in flat‐bottom 96‐well microtiter plates. Culture supernatants were harvested and analyzed at 24 h for IL‐2 (BioLegend, California, United States; Cat. Number 431004), IL‐4 (BioLegend, CA, USA), and IL‐12p40 (eBioscience, CA, USA); 48 h for IL‐22 (eBioscience, CA, USA); 72 h for IL‐10 (eBioscience, CA, USA), IL‐17A (eBioscience, CA, USA), IL‐17F (BioLegend, CA, USA), and IL‐23 (eBioscience, CA, USA); and 96 h for IFN‐γ (eBioscience, CA, USA) and IL‐12(p70) (BioLegend, CA, USA) using commercial ELISA kits according to the manufacturer’s instructions (Biolegend, CA, USA). The data represent the mean of five independent experiments.

### 2.9. CTL Cytotoxicity Activity Assay

Following the previously reported method [20], CytoTox 96 Nonradioactive Cytotoxicity Assay Kits (Promega, WI, USA) were used to measure CTL activity. Briefly, spleen‑derived effector cells were coincubated with 100 U/mL of murine IL‑12 (eBioscience, CA, USA), and in order to detect *T. gondii*–specific CTL induced by pVAX‐GRA47, pVAX‐GRA72, or pVAX‐GRA47 + pVAX‐GRA72, Sp2/0 mouse cells were introduced with the plasmids and served as target cells following a 5‑day period. Effector and target cells were then combined for 6 h at effector/target ratios of 10, 20, 40, and 80 to 1. Specific cell lysis was determined according to the equation:

Experimental− Effector spontaneous− Target spontaneous/Target maximum− Target spontaneous×100.

### 2.10. Statistical Evaluation

All statistical evaluations were carried out using GraphPad Prism 8 (San Diego, CA, USA). To compare the experimental groups, a one‑way ANOVA with Tukey’s post hoc test was applied. Both Kaplan–Meier analysis and the log‐rank test were used to evaluate survival outcomes. Significance was defined as *p* < 0.05.

## 3. Results

### 3.1. Bioinformatic Analysis of Antigenic Properties and Identification of Recombinant Plasmids In Vitro

**Figure 1:**
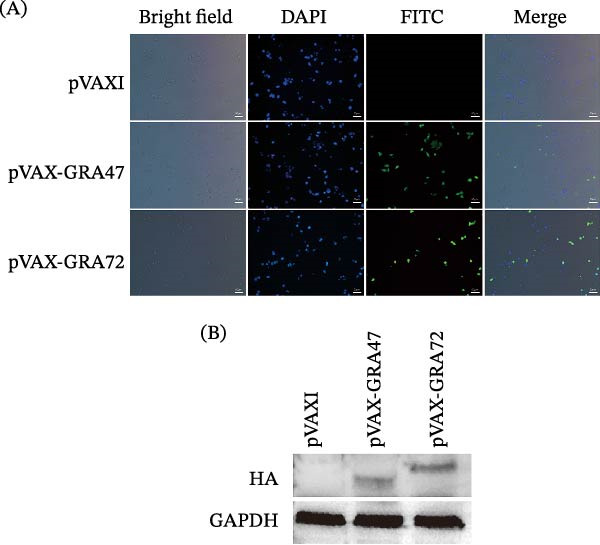
Analysis of recombinant TgGRA47 and TgGRA72 protein expression in 293‐T cells. (A) Immunofluorescence assay of 293‐T cells introduced with empty pVAX I, pVAX‐GRA47, or pVAX‐GRA72. Recombinant proteins were detected using an anti‐HA polyclonal antibody (green). Nuclei were counterstained with DAPI (blue). Brightfield images and merged images are provided to show cellular morphology and protein localization. Scale bars = 50 μm. (B) Western blot analysis of recombinant protein expression.

Bioinformatic analysis indicated that TgGRA47 and TgGRA72 contain multiple regions with high antigenic index, strong hydrophilicity, and high surface probability. These regions also corresponded to flexible structural domains, suggesting potential accessibility for B‐cell recognition. These results support the potential immunogenicity of TgGRA47 and TgGRA72 as vaccine antigens (Figure S1). To assess the in vitro expression of pVAX‑GRA47 and pVAX‑GRA72, IFA was used to confirm the green fluorescence under the fluorescence microscope. As shown in Figure 1, green fluorescence, specifically localized, was seen in HEK 293‑T cells upon transfection with pVAX‑GRA47 or pVAX‑GRA72. No fluorescence, however, was observed in cells transfected with the empty pVAX I. Nuclear counterstaining with DAPI confirmed the cellular localization of the expressed proteins (Figure 1A). Further evidence from Western blot analysis confirmed recombinant protein synthesis in HEK 293‑T cells introduced with pVAX‑GRA47 alone or pVAX‑GRA72. Two specific bands corresponding to TgGRA47 and TgGRA72 were detected, while no distinct band was detected in cells receiving the blank pVAX I plasmid (Figure 1B).

### 3.2. Humoral Immunity

**Figure 2:**
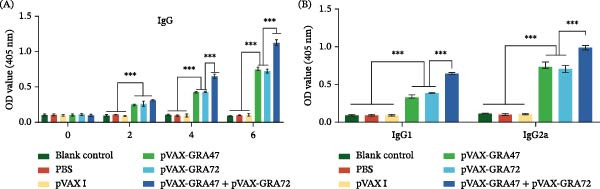
Evaluation of *T. gondii*–specific antibody responses triggered by DNA vaccination using either one or several genes. (A) Levels of anti‐*T. gondii* serum IgG levels in BALB/c mice at 0, 2, 4, and 6 weeks after immunization, measured by ELISA using sera diluted at 1:100. (B) Levels of anti‐*T. gondii* IgG1 and IgG2a responses measured 2 weeks after the booster dose, measured at sera dilution of 1:100. *n* = 6;  ^∗∗∗^ *p* < 0.001. Data are presented as the means ± SD.

Serum samples were harvested from both naïve and immunized mice at weeks 0, 2, 4, and 6 after DNA vaccination with pVAX‑GRA47 and/or pVAX‑GRA72. ELISA was then performed on these samples to quantify specific total IgG and IgG isotypes (IgG1 and IgG2a). Figure 2A illustrates that the IgG titers in all immunized groups were markedly elevated than the values from the three control groups (*p* < 0.001). The multigene group induced the highest levels of IgG, exhibiting a marked elevation relative to both the group of pVAX‐GRA47 and pVAX‐GRA72 (*p* < 0.001). However, no significant difference was observed between these two single‐gene groups (*p* > 0.05). Also, the levels of IgG rose progressively in all vaccinated groups, reaching their peak 6 weeks after the last immunization. By contrast, the three control groups showed no marked elevation in antibody titers (*p*  > 0.05). In similar with the increased levels of IgG, the concentrations of both the levels of IgG1 and IgG2a and the proportion (IgG2a/IgG1) were the highest in the group of DNA immunization with pVAX‐GRA47 and pVAX‐GRA72, and a single gene immunization with pVAX‐GRA47 or pVAX‐GRA72 is intermediate (Figure 2B). These results suggest that DNA vaccines induced a Th1‐biased humoral immune milieu, and this immune polarization is consistent with the cellular immune responses observed in subsequent lymphocyte proliferation, flow cytometry, and cytokine analyses.

### 3.3. Cytokine Production

**Figure 3:**
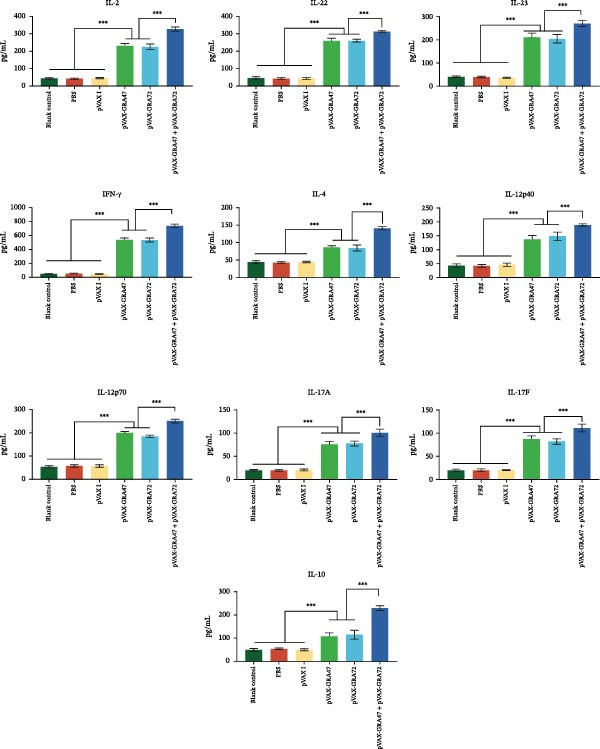
Cytokine levels in splenocytes from mice vaccinated with pVAX‑GRA47 and/or pVAX‑GRA72. *n* = 6;  ^∗∗∗^ *p* < 0.001. Data are presented as the means ± SD. pVAX‐GRA47 and/or pVAX‐GRA72.

ELISA was employed to measure cytokine levels in splenocyte cultures obtained from mice that had or had not received immunization, collected at 2 weeks postbooster, in order to determine cytokine production across the groups. As can be seen in Figure 3, DNA vaccination with pVAX‐GRA47 and pVAX‐GRA72 induced significantly higher levels of Th1‐associated cytokines, with IFN‐γ, IL‐2, and IL‐12 as well as Th‐17 associated cytokines, namely, IL‑17A, IL‑17F, IL‑22, and IL‑23, when contrasted with the two single‑gene groups (pVAX‑GRA47 and pVAX‑GRA72) (*p* < 0.001), while no significant difference was observed between the two single‐gene groups (*p* > 0.05). Consistent with the profiles of these Th1/Th17 cytokines, the levels of Th2‐type cytokines IL‐4 and IL‐10 were also significantly upregulated in splenocyte cultures from the multigene group than in both single‐gene groups and the three control groups (*p* < 0.001). In contrast, no significant difference was observed between the two single‐gene groups (*p* > 0.05), and similarly, no significant differences were found among the three control groups (*p* > 0.05).

### 3.4. Analysis of T Cell Responses and DC Activation

**Figure 4:**
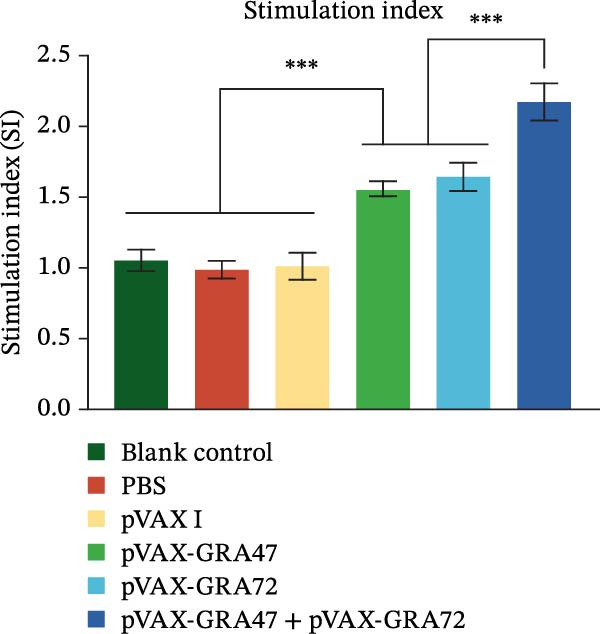
Lymphocyte stimulation index (SI)–based proliferative responses of splenocytes from control and single‑/multigene immunized mice. *n* = 6;  ^∗∗∗^ *p* < 0.001. Data are presented as the means ± SD.

Following by stimulation with TLA or ConA, to determine lymphocyte proliferation, the MTT assay was used. As demonstrated in Figure 4, all immunized groups showed a higher SI in splenocyte cultures than the levels found in unimmunized controls. Combined administration of pVAX‐GRA47 and pVAX‐GRA72 induced the highest SI unlike the control groups.

**Figure 5:**
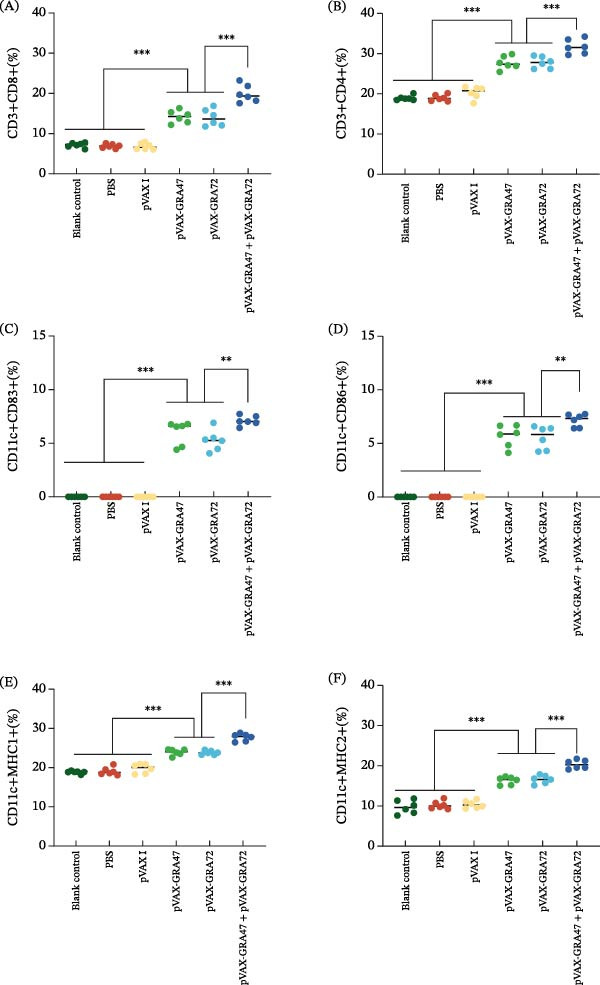
Flow cytometry analysis of splenic lymphocytes in vaccinated and control mice. The proportions of CD4^+^ or CD8^+^ T cells in vaccinated (A) and control mice (B). The percentage of CD83 (C) and CD86 molecules (D) on splenic DCs. The percentage of MHC‐I (E) and MHC‐II molecules (F) on splenic DCs. *n* = 6;  ^∗∗∗^ *p* < 0.001. Data are presented as the means ± SD.

For further characterization of the cell‑mediated immune reaction, the proportions of CD4^+^ and CD8^+^ T cells isolated from splenocytes of every experimental group were assessed using flow cytometry (Figure S2). Consistent with earlier results, a markedly elevated proportion of CD8^+^ T (Figure 5A) and CD4^+^ T cells (Figure 5B) was seen in the multiplasmid vaccination groups versus the groups that received a single‑gene construct (*p* < 0.001), while T‐cell frequencies were comparable between the two single‐gene groups (*p* > 0.05).

Similarly, flow cytometry was also used to analyze the expression of surface markers CD83, CD86, MHC‐II, and MHC‐I in DCs (Figure S1). As shown in Figure 5C,D, the levels of CD83 and CD86 were markedly elevated in the dual‑plasmid group (pVAX‑GRA47 + pVAX‑GRA72) relative to the single‑plasmid groups (pVAX‑GRA47 or pVAX‑GRA72) (*p* < 0.001). Additionally, higher levels of MHC‑I (Figure 5E) and MHC‑II (Figure 5F) molecules were noted in immunization of mice with a DNA vaccine cocktail, as opposed to immunization with a single‑gene plasmid (*p* < 0.001), whereas the differences between the two single‐gene groups, as well as among the three control groups, did not reach statistical significance (*p* > 0.05).

**Figure 6:**
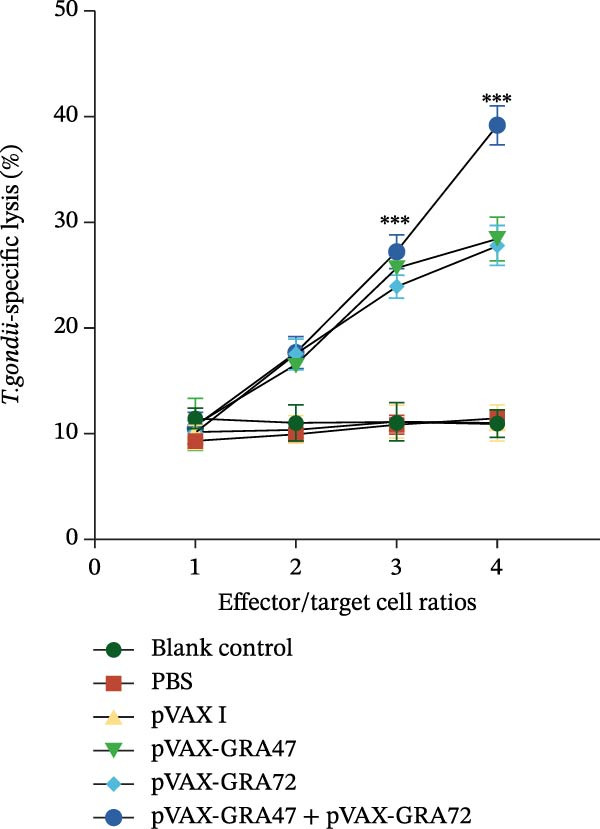
CTL responses of splenic lymphocytes from vaccinated mice. The *x*‐axis represents effector‐to‐target ratios, while the *y*‐axis shows the percentage of *T. gondii*–specific cell lysis.*n* = 6;  ^∗∗∗^ *p* < 0.001. Data are presented as the means ± SD.

Also, CTL responses were also activated after vaccination via pVAX‐GRA47 or/and pVAX‐GRA72. Figure 6 illustrates that splenic CTL activity in all vaccinated mice rose steadily with increasing effector‑to‑target cell ratios, peaking at 80:1. Also, DNA vaccination with pVAX‐GRA47 and pVAX‐GRA72 have induced higher CTL activity than that observed in mice receiving either the single‑gene plasmid pVAX‑GRA47 or pVAX‑GRA72 (*p* < 0.001). However, no significant differences were detected between the two single‐gene groups or among the three groups used as controls (*p* > 0.05).

### 3.5. Effectiveness of Protection Against *T. gondii* in Mice

**Figure 7:**
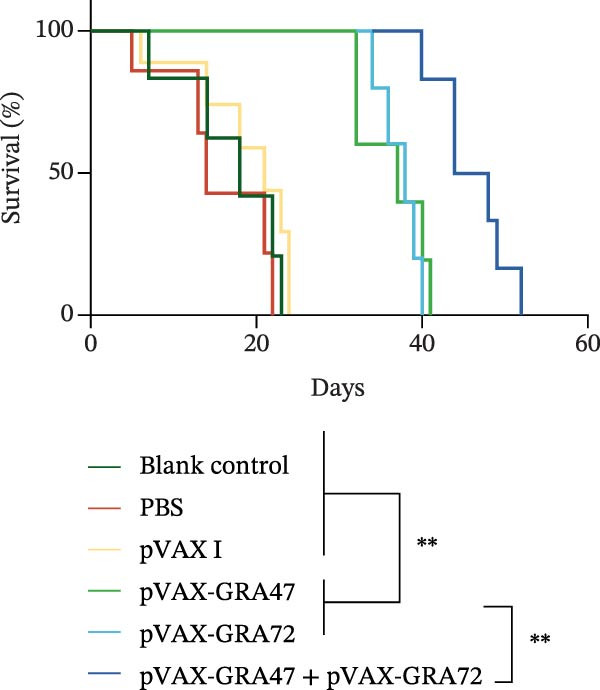
Survival of immunized mice was monitored 2 weeks following the last vaccination. BALB/c mice were challenged with 100 PRU strain cysts, and their survival rates were determined. *n* = 10;  ^∗∗^ *p* < 0.01. Data are presented as the means ± SD.

Two weeks following the last vaccination with pVAX‑GRA47 and/or pVAX‑GRA72, BALB/c mice were challenged with the *T. gondii* PRU strain to assess the protective efficacy. The challenge was administered either as a lethal dose of 100 cysts or a nonlethal dose of 20 tissue cysts. As shown in Figure 7, the mice showed a significantly prolonged survival time in all immunized groups. In contrast, all unimmunized mice exposed to 100 cysts of the *T. gondii* PRU strain succumbed by day 23 postinfection. Cocktailed with pVAX‐GRA47 and pVAX‐GRA72 induced a longer survival time compared to DNA immunization with a single gene coding GRA47 or GRA72 (*p* < 0.01).

**Figure 8:**
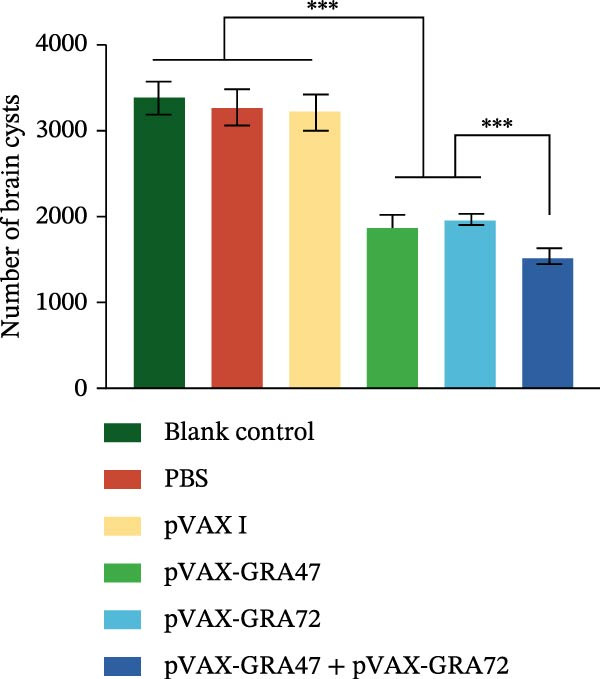
Protection against chronic toxoplasmosis was evaluated in immunized mice 2 weeks after the final booster. Data are means ± SD (representative of three experiments). *n* = 6;  ^∗∗∗^ *p* < 0.001, compared with the control groups.

Also, all immunized mice are able to reduce the number of brain cysts compared to the control groups (*p* < 0.001), with reduction rates of 37.6% and 38.3% in the pVAX‐GRA47 and pVAX‐GRA72 groups, while the two single‑gene groups did not differ significantly (*p* > 0.05). A significantly higher reduction (53.2%) was achieved in the pVAX‐GRA47 and pVAX‐GRA72 group compared to both single‐gene groups (*p* < 0.001) (Figure 8).

## 4. Discussion

DNA vaccines have recently been recognized to be a promising approach to prevent animals and humans from infection with bacterial, viral, and even parasitic infection, including intracellular parasites [13, 24]. Also, several commercial DNA vaccines have been licensed and explored for combating infectious diseases [17, 25]. Due to the priority of low cost and easy production of DNA vaccines, together with its abilities of eliciting long‐lasting humoral and cellular immune responses, the development of DNA vaccines has become a promising alternative used for fighting against toxoplasmosis [15]. Although the considerable studies have been devoted to the identification of DNA vaccine candidates, no DNA vaccine candidates have been demonstrated to be able to induce sterilizing immunity to completely prevent infection with *T. gondii* in mouse models. Hence, identifying novel vaccine candidates continues to be a dynamic research field and is viewed as a crucial strategy for advancing DNA vaccination against toxoplasmosis.

Some *T. gondii* GRA proteins have previously been identified to be DNA vaccine candidates, including GRA15, GRA16, GRA24, and GRA25, which have been shown to be able to provide considerable protection against both acute and chronic infection with *T. gondii* in experimental animals [26–31]. In this study, DNA vaccines expressing TgGRA47 or TgGRA72 were generated and then assessed for their immunogenicity and protective efficacy in mouse models. The mice were challenged with either 100 cysts (lethal) or 20 tissue cysts (nonlethal) of the PRU strain. Our results revealed that vaccination with pVAX‑GRA47 or pVAX‑GRA72 triggered both humoral and Th1/Th17‑type cellular immune responses, leading to prolonged survival following challenge with 100 *T. gondii* PRU cysts and diminished brain cyst numbers after challenge with the same parasite. Especially, the cocktail of pVAX‑GRA47 and pVAX‑GRA72 conferred superior protective immunity relative to single‑gene–based DNA immunization, which is in line with investigations into multiantigen DNA immunization [32, 33], suggesting that a DNA vaccine candidate encoding multigene could significantly strengthen the immunity induced by vaccination with a single‑gene construct.

It is well established that antibody‑mediated humoral immunity plays a critical role in protection against *T. gondii* infection [34]. The neutralization of parasite attachment to host cells can be achieved by specific antibodies, which act via antibody‑mediated activation of the classical complement pathways [35, 36]. The analysis of IgG subclasses is commonly used as an indicator of immune polarization. In general, IgG2a is associated with Th1‐type responses, whereas IgG1 is linked to Th2‐type immunity [37]. In the present study, specific anti‑*T. gondii* IgG titers and IgG isotypes were evaluated in vaccinated as well as unvaccinated mice, which aligns with observations from other multiantigen DNA vaccine studies [23], in combination with pVAX‐GRA47 and pVAX‐GRA72 markedly increased the levels of total IgG, IgG1, and IgG2a, which suggests that the DNA vaccines may promote a Th1‐biased immune milieu, which is generally favorable for protection against intracellular pathogens such as *T. gondii*.

As professional antigen‐presenting cells, DCs play a crucial role in initiating both innate and adaptive immunity against *T. gondii* infection, along with the increased expression of CD83, CD86, MHC‐I, and MHC‐II markers on splenic DCs [38]. Also, DCs play a significant function in mediating T‐cell killing of intracellular pathogens, including *T. gondii*, by means of surface markers, CD83 and CD86 acting on T cells, for example, CD86 can bind to CD28 molecule on the surface of T cells, providing a costimulatory signal to the activation of T cells, and CD83 plays an important role in regulating of T cell stimulation [39]. In addition, MHC‐II molecules expressed by mature DC mainly activate CD4+T cells through the presentation of exogenous, and MHC‐I molecules expressed in all nucleated cells could promote the activation of CD8+T cells through the way that present endogenous antigen to the cell surface [40, 41]. In the present study, DNA immunization with pVAX‐GRA47 and or pVAX‐GRA72 significantly increased the expression of CD83, CD86, MHC‐I, and MHC‐II on splenic DC, suggesting enhanced maturation and activation of these antigen‐presenting cells. Upregulation of costimulatory molecules and MHC complexes is widely recognized as a hallmark of DC maturation and an essential prerequisite for efficient T‐cell priming [42]. These changes may facilitate antigen presentation to CD4+ and CD8+ T cells, leading to subsequent T‐cell activation. Activated T lymphocytes, especially CD8+T cells, can further differentiate into CTLs that exert immunotoxicity against the parasite [43]. However, it should be noted that although increased expression of CD83, CD86, MHC‐I, and MHC‐II indicates enhanced DC maturation, the presence of these markers alone does not directly demonstrate improved antigen‐presenting capacity. Functional assays evaluating antigen‐specific T‐cell activation would be required to definitively confirm the antigen‐presentation efficiency of DCs following vaccination.

In common, Th1‐mediated immunity is necessary for killing intracellular parasites, involving the secretion of cytokines IFN‐γ, IL‐12, and IL‐2. IFN‐γ, the most important cytokine production by Th1‑type lymphocytes, induces an inflammatory response, promoting the efficient elimination of *T. gondii* tachyzoites [44]. A further Th1‑oriented cytokine, IL‐2, is also necessary for resistance against *T. gondii* infection, which depends upon its regulation of the multiplication and functional responses of cytotoxic T cells [45, 46]. Besides, IL‐12, including IL‐12p70 and IL‐12p40, is usually generated by DCs, macrophages, and neutrophils, and it could also in turn induce differentiation and proliferation of Th1‐type T cells, including memory T cells, leading to the production of IFN‐γ by natural killer cells and T cells [47]. In the present study, significantly upregulated levels of IFN‐γ, IL‐2, IL‐12p40, and IL‐12p70 were detected in immunized groups. These findings indicate that vaccination using the DNA plasmids pVAX‑GRA47 and/or pVAX‑GRA72 triggers a robust Th1‑skewed immune reaction. Such a Th1‐dominant immune environment is known to promote macrophage activation and CTL‐mediated cytotoxic responses, which are essential effector mechanisms for limiting intracellular parasite replication. Therefore, the enhanced Th1 responses observed in immunized mice may contribute to controlling parasite proliferation and dissemination, thereby partially explaining the prolonged survival and reduced parasite burden observed after the challenge with the *T. gondii* PRU strain.

In the meanwhile, both IL‐4 and IL‐10, the marker cytokines for Th2 cells, are also elicited in the immunized mice, which are able to play a modulatory function of an excessive Th1‑type response through limitation of both the induction of inflammation and the inhibition of severe immunopathology caused by CD4+ T cells [48]. Thus, the coexistence of Th1‐ and Th2‐associated cytokines may help maintain an immunological balance that supports effective parasite control while limiting immune‐mediated tissue damage.

Recently, Th17 cells are presented to play an antimicrobial role in mediating host protection against intracellular parasites, including *Trypanosoma cruzi*, *Plasmodium* spp., and *Cryptosporidium parvum* [49, 50]. Also, Th17 cells are termed as IL‐17‐producing T cells, and they could secrete a set of cytokines, such as IL‐17 (L‐17A and IL‐17F), IL‐22, and IL‐23, which are considered to be inflammatory cytokines [51, 52]. Th17 cells can act on the control of *T. gondii* infection in the early stages, involved in the accumulation and activation of neutrophils, leading to the induction of neutrophilic inflammation [53]. Among these Th17‐associated cytokines, IL‐17 is the most important for the resistance to intracellular parasitic infections, via inducing the expression of the stimulating factor granulocyte macrophage‐colony, IL‐1β, IL‐6, IL‐8, and TNF [54]. Consistent with these observations, the present study showed significantly higher amounts of IL‑17A, IL‑17F, IL‑22, and IL‑23 observed in immunized animals, indicating an activation of Th17‐associated immune responses, which corresponds to previous findings in effector responses induced by the *Mycobacterium* tuberculosis and *Leishmania donovani* vaccines [55, 56]. The activation of Th17‐mediated pathways may facilitate early innate immune responses and contribute to limiting parasite spread within the host. Importantly, the coordinated induction of Th1‐ and Th17‐type immune responses recorded here provides a potential immunological explanation for the protective efficacy of the DNA vaccines, as these responses collectively promote the activation of macrophages, cytotoxic T cells, and neutrophils that restrict intracellular parasite replication and dissemination.

T‐cell–mediated adaptive immunity plays a vital role in the resistance to primary *T. gondii* acute infection and even the reactivation of chronic toxoplasmosis [34]. Following the *T. gondii* invasion, CD8+ T lymphocytes are critical to limit the tachyzoites spreading in the early phase of acute *T. gondii* infection, and even CD8+ T lymphocytes play a key role in mediating lysis of brain cysts through their cytolytic activity during the late stage of chronic infection with *T. gondii*, cooperatively with CD4+ T lymphocytes [57–59]. Moreover, CTLs, especially CD8+ CTL activity, are key to control intracellular *T. gondii* replication, and also the activated specific CTL response has been considered to be an important prerequisite for the creation of an effective *T. gondii* vaccines [60]. In the present study, spleen lymphocytes have shown a significant splenocyte proliferation and also activated CTL activity in the immunized mice, suggesting that DNA immunization with these candidates induced an activation of *T. gondii*–specific CTL, contributing to the control of this intracellular parasite. Flow cytometric analysis revealed markedly elevated proportions of CD4+ and CD8+ T cells in immunized mice, particularly in those receiving the multigene vaccine. These results suggest that DNA vaccination with pVAX‑GRA47 and/or pVAX‑GRA72 induces effector T‑lymphocyte responses, potentially contributing to reduced brain cyst formation and restricted dissemination of *T. gondii*.

Some previous studies have been devoted to evaluating the protective efficacy of the DNA vaccine against the *T. gondii* RH (Type I) strain, which is highly virulent and the lethal *T. gondii* parasite strain in mouse models [61, 62]. Also, a high dosage of the RH strain is considered to be a failed observation of a longer survival time. However, Type II strain (e.g., PRU strain) is the predominant lineage causing toxoplasmosis in humans, and also the challenge of 80–100 cysts of strain PRU per mouse intragastrically is more suitable to observe survival time after immunization with vaccine candidates [63, 64]. Therefore, in our study, we have found that vaccination using pVAX‐GRA47 and/or pVAX‐GRA72 markedly extended the survival period after challenge with 100 cysts (acute infection) and also reduced the formation of brain cysts, following by chronic infection with 20 cysts. Consequently, this DNA immunization has only elicited partial protective efficacy, as well as eventual death and clinical signs during the late infection phase. Thus, further studies should consider incorporating molecular adjuvants to prolong survival and/or reduce the cyst burden. Furthermore, we should also consider the long‐term stability and safety of recombinant DNA plasmids encoding TgGRA47 and TgGRA72 targeting *T. gondii* infection, thus indicating its promise as a feasible approach for managing toxoplasmosis in human and animal populations.

## 5. Conclusions

In conclusion, our study demonstrated both DNA vaccines encoding TgGRA47 and/or TgGRA72 are potential vaccine candidates, exhibiting activated antibody‑mediated immunity as well as Th1‑ and Th17‑polarized responses, generating a strong protective immune response against toxoplasmosis in experimental mice. These observations may contribute to the generation of more potent vaccines targeting *T. gondii* infection and might also enhance protection against additional apicomplexan parasites.

## Author Contributions

Conceptualization: Zihan Zhou and Jia Chen. Methodology: Zihan Zhou and Jingqi Mu. Software: Zihan Zhou and Jingqi Mu. Validation: Zihan Zhou, Jingqi Mu, and Jia Chen. Formal analysis: Zihan Zhou, Jie Sun, Chenhui Zhou, and Jia Chen. Writing – original draft preparation: Zihan Zhou and Jingqi Mu. Supervision: Jia Chen. Project administration: Chenhui Zhou and Jia Chen. Funding acquisition,: Chenhui Zhou and Jia Chen.

## Funding

This research was funded by the Ningbo Science and Technology Innovation 2025 Major Project (Grant 2022Z125), the Ningbo Top Medical and Health Research Program (Grant 2022020304), and the Natural Science Foundation of Zhejiang Province, China (Grant LY22C180004).

## Disclosure

All authors have read and agreed to the published version of the manuscript.

## Conflicts of Interest

The authors declare no conflicts of interest.

## Supporting Information

Additional supporting information can be found online in the Supporting Information section.

## Supporting information


**Supporting Information** Figure S1: Bioinformatic prediction of GRA47 and GRA72. Figure S2: Gating strategies used to identify CD8⁺ T cells, CD4⁺ T cells, and dendritic cells in the spleen.

## Data Availability

The data presented in this study are included in the article. Further inquiries can be directed to the corresponding authors.
