# Supplementary material for: Protective Immunity Induced by DNA Vaccines Encoding TgGRA47 and TgGRA72 Against Toxoplasma gondii Infection in BALB/c Mice
Source: Transbound Emerg Dis. 2026 Jun 5;2026:9513737. doi: 10.1155/tbed/9513737 (PMC13238239; doi:10.1155/tbed/9513737)
Supplement: Supplementary file 1 — Supporting Information Figure S1: Bioinformatic prediction of GRA47 and GRA72. Figure S2: Gating strategies used to identify CD8⁺ T cells, CD4⁺ T cells, and dendritic cells in the spleen. [file TBED-2026-9513737-s001.docx]

**Supplementary material**


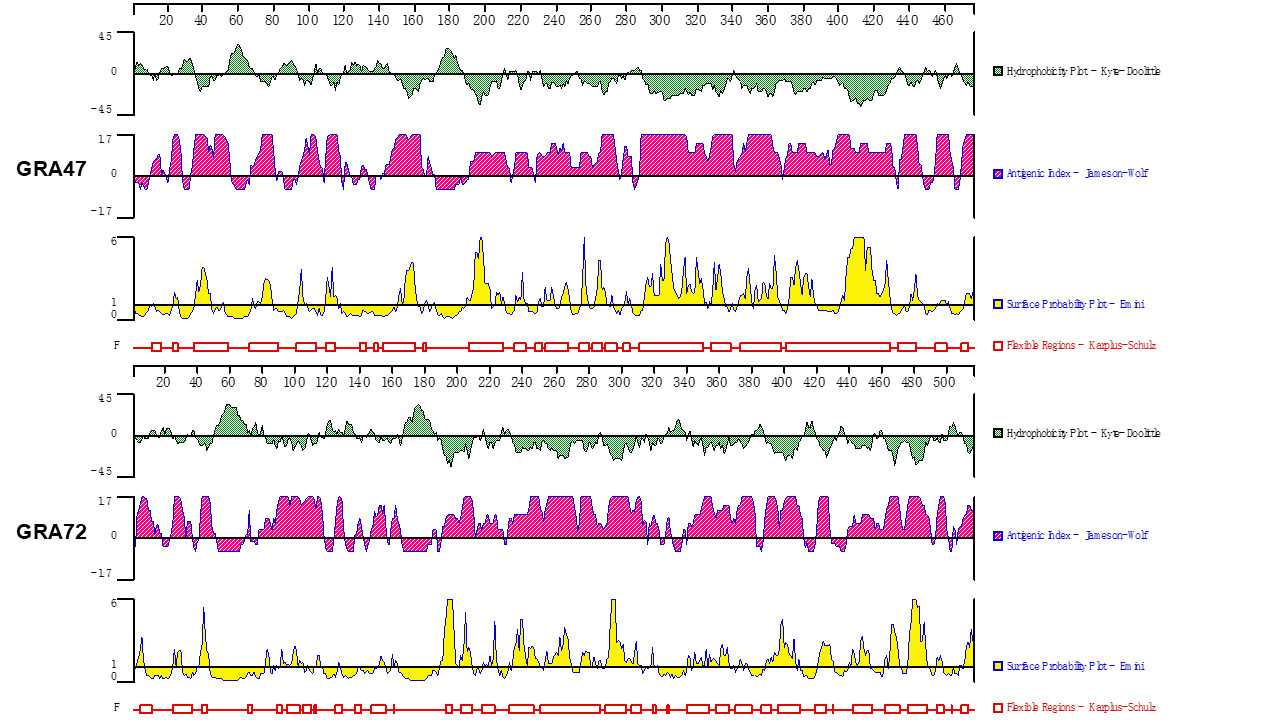


**Supplementary Figure 1..** Bioinformatic Prediction of GRA47 and GRA72.


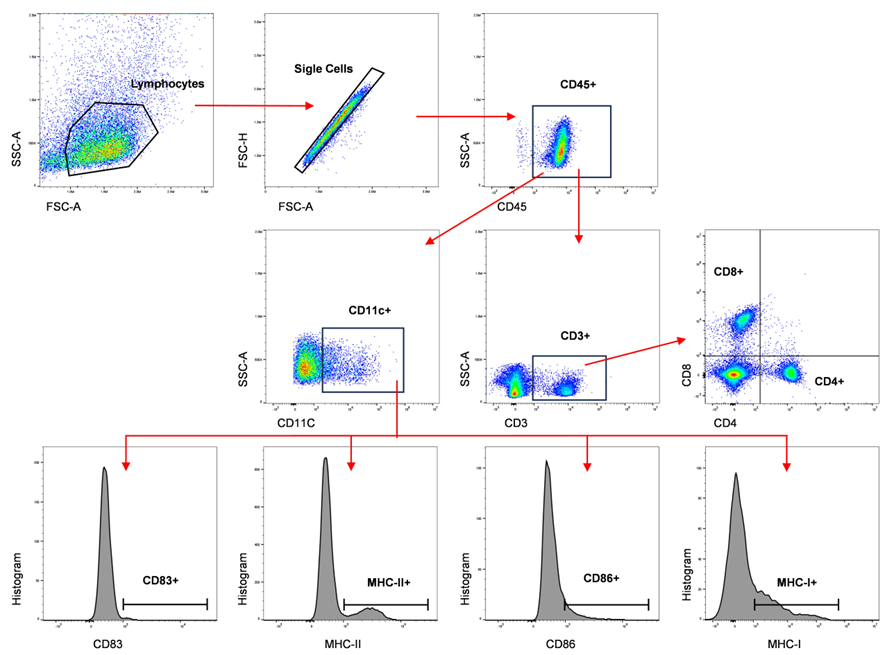


**Supplementary Figure 2.** Gating strategies for CD8+ T cells, CD4+ T cells and dendritic cells in the spleen.
